# Effectiveness of substance use disorder treatment as an alternative to imprisonment

**DOI:** 10.1186/s12888-024-05734-y

**Published:** 2024-04-09

**Authors:** Suvi Virtanen, Mikko Aaltonen, Antti Latvala, Mats Forsman, Paul Lichtenstein, Zheng Chang

**Affiliations:** 1 Department of Medical Epidemiology and Biostatistics, Karolinska Institutet, 171 77 Stockholm, Sweden; 2 School of Educational Sciences and Psychology, University of Eastern Finland, Joensuu, Finland; 3 UEF Law School, University of Eastern Finland, 80101 Joensuu, Finland; 4 Institute of Criminology and Legal Policy, University of Helsinki, 00014 Helsinki, Finland

**Keywords:** Substance use disorder, Treatment, Drug court, Harm reduction, Crime, Incarceration, Recidivism, Mental health

## Abstract

**Introduction:**

Drug courts are criminal justice programs to divert people with substance use disorders from incarceration into treatment. Drug courts have become increasingly popular in the US and other countries. However, their effectiveness in reducing important public health outcomes such as recidivism and substance-related health harms remains ambiguous and contested. We used nationwide register data from Sweden to evaluate the effectiveness of contract treatment sanction, the Swedish version of drug court, in reducing substance misuse, adverse somatic and mental health outcomes, and recidivism.

**Methods:**

In this prospective cohort study, two quasi-experimental designs were used: difference-in-differences and the within-individual design. In the latter, we compared the risk of outcomes during time on contract treatment to, 1) parole after imprisonment and, 2) probation.

**Results:**

The cohort included 11,893 individuals (13% women) who underwent contract treatment. Contract treatment was associated with a reduction of 7 percentage points (95% CI: -.088, -.055) in substance misuse, 5 percentage points (-.064, -.034) in adverse mental health events, 9 percentage points (-.113, -.076) in adverse somatic health events, and 3 fewer charges (-3.16, -2.85) for crime in difference-in-differences analyses. Within-individual associations suggested that the same individual had longer times-to-event for all outcomes during contract treatment than on parole or on probation.

**Conclusions:**

Contract treatment is an effective intervention from both public health and criminal justice perspective. Our findings suggest that it is a superior alternative to incarceration in its target group. Further, we find that an implementation approach that is less punitive and more inclusive than what is typical in the US can be successful.

**Supplementary Information:**

The online version contains supplementary material available at 10.1186/s12888-024-05734-y.

## Introduction

Drug courts are popular criminal justice programs that aim to approach criminal offending from a public health perspective. Instead of punishment, their purpose is to address substance misuse as the underlying driver of criminal behavior by incentivizing individuals with a substance use disorder into treatment with an agreement that charges against them will be reduced upon treatment completion. The programs in the US typically involve frequent urine testing, treatment attendance, and appearance before the court for status hearings [1, 2]. Drug courts enjoy bi-partisan political support in the US and have been accepted as an important part of the criminal justice system in other countries as well.

Despite their popularity, the empirical evidence for their effectiveness remains modest. Meta-analyses of drug court evaluations found that they may reduce recidivism [3–5] but only a minority of included studies were methodologically rigorous [3]. The main limitations of prior studies were short follow-ups that often overlapped completely with time in treatment, inappropriate control groups, and insufficient adjustment for confounding. Moreover, drug courts have been promoted as being more of a public health rather than criminal justice-based response to substance use problems [6, 7]. The public health view on substance use has begun to place increasing emphasis on harm reduction, which aims to lessen the negative health consequences associated with alcohol or drug use [8, 9]. There is little evidence that drug courts achieve this goal, because hardly any drug court evaluations have assessed health outcomes of participants [3, 10–12].

Critics of drug courts point out that besides having weak evidence base in terms of being effective in reducing recidivism, the US drug courts have many inherently problematic aspects [13–18]. A large proportion of drug courts deem individuals who have committed a violent crime or crimes involving sales of narcotics, at any point in time, as ineligible for participating [3, 19, 20]. In effect, drug courts tend to target “low-risk” individuals charged with purchasing or possessing illicit drugs, who do not necessarily have an actual substance use disorder [21, 22], while excluding those with more serious criminal history who would, according to the risk-need-responsivity principles, most likely benefit from treatment [23, 24]. Many drug courts require that participants pay fees [25–27], and failure to pay can result in sanctions or dismissal [26, 27]. They also regularly deny access to medication-assisted treatment to people with opioid use disorder, despite this treatment being the gold standard [28, 29]. The judges’ notable discretion over rewards and sanctions posed on clients has also been criticized [16, 17]. In a typical US drug court, the judge not only retains his authority to set the terms of treatment, but also assumes the role of regulating it [13]. The court, rather than the treatment center, becomes the focal point of the treatment process [16, 17]. For instance, in situations where a client relapses, which is a normal and expected part of the recovery process, the judge can use temporary incarceration as a punishment [27, 30].

There are differences in the way the drug court model is implemented in different countries. Sweden established a policy similar to drug court in the late 1980s, but it has major differences compared to its US counterpart. For instance, cases involving violent crimes and more serious drug-related crimes are not automatically excluded from eligibility, and there is no judge oversight over treatment or court appearances where the client’s treatment progress is praised or castigated. The effectiveness of the Swedish contract treatment has not been evaluated, so it remains unclear an implementation approach that is less punitive and more inclusive than what is typical in the US can produce positive outcomes.

We used nationwide administrative register data from Sweden to evaluate the effectiveness of contract treatment in reducing substance misuse, adverse somatic and mental health outcomes, and criminality. We conducted the evaluation using two quasi-experimental study designs—difference-in-differences and the within-individual design—to improve internal validity compared to previous observational drug court evaluations. In the latter design, we compared the risk of outcomes during time on contract treatment to time (1) under parole after imprisonment, and (2) regular probation without a treatment component. Triangulation of evidence, i.e., the use of different methodological approaches that have their own sets of assumptions and limitations, provides more rigorous evidence than relying on a single approach alone.

## Methods

### Study cohort

We identified persons who initiated contract treatment between years 1999 and 2012 from the Swedish Prison and Probation Service’s client register. A detailed description of contract treatment sanction is available in the Supplement. While the sanction is intended mainly for people with a substance use disorder, it is sometimes offered to individuals who need treatment for other problems (e.g., sexual paraphilia, gang involvement) that contribute to their criminal behavior. For this reason, we excluded sanctions where the primary offense was a sexual offense or related to firearms. All individuals born in or immigrating to Sweden are assigned a unique personal identity number. We used de-identified personal identity numbers of cohort members to link information on the outcomes and cohort characteristics from other register sources. The register linkage was approved by the Swedish Ethical Review Authority. Informed consent to participate was not obtained from all the participants in the study. Informed consent requirement was waived by the Swedish Ethical Review Authority because the study was register-based, and data were anonymized.

### Difference-in-differences approach

Difference-in-differences is a quasi-experimental study design that compares changes in outcomes over time between a population that becomes enrolled in a treatment (the treatment group) and a population which does not (the control group). We used a version of difference-in-differences which exploits variation in treatment timing, and uses *not-yet-treated* individuals as controls. In other words, for individuals treated at time *t*, we used individuals treated at *t* + ^*1,2,..n*^ who were also observed at time *t-1* and *t*, as controls to estimate the treatment effect at time *t*. Causal target estimand is the average treatment effect on the treated (ATT). Assumptions to identify ATT include parallel trends (in the absence of treatment, the difference in outcome between the treatment and control group remains constant over time), no anticipation of treatment (future treatments do not affect current outcomes), and the stable unit treatment values assumption (SUTVA; no interference or several versions of treatment).

### Follow-up

For each individual, the follow-up started 2 years before the start date of contract treatment sanction. Sanction start date was replaced with the date of conviction in situations where the conviction date occurred earlier than the sanction start date; this was done to reduce the risk of violating the “no anticipation of treatment” assumption. While individuals are likely aware of the possibility of receiving contract treatment before conviction, the final decision on whether contract treatment is granted is made by the judge after trial. The date of conviction was the same as the start of sanction in 90% of cases. Follow-up ended 2 years after the sanction start date, at death, emigration from Sweden, entry to prison, or December 31st 2013, whichever occurred first. We structured the dataset as an unbalanced long panel where follow-up time was aggregated by quarter year (Figure S1). If an individual had undergone contract treatment more than once, the first treatment was selected for the analysis.

### Measures

Outcomes included acute substance misuse events, acute mental health events, acute somatic health events, and being suspected of a crime by the police. The outcomes were investigated separately. The three medical outcomes were retrieved from the National Patient Register, which covers publicly funded inpatient and outpatient health care nationwide. ICD-10 codes used to define the outcomes and cohort characteristics are reported in Tables S1 to S4. Only diagnoses received during unplanned (“emergency”) inpatient or outpatient visits were included, and diagnoses received during planned visits (e.g., follow-ups and referrals) were excluded. We aimed to exclude outcomes that reflect increased service use (e.g., positive outcomes such as attending outpatient clinic regularly as part of psychiatric treatment), rather than outcomes that the treatment is expected to reduce (e.g., overdose, hospitalization for mental health emergency). Crime outcomes were collected from the Register of Persons Suspected of Offences, which includes people who are suspected of crime after a completed investigation by police, customs authority, or prosecution service. We used the date when the crime was committed to determine the timing of the outcome. Outcome was defined as having been suspected of any type of crime.

### Statistical analyses

We estimated dynamic treatment effects, showing how the effect of treatment changes as time progresses (an “event study”). We used the linear probability model for diagnosis-based outcomes; the outcome variable was binary (1 = diagnosis, 0 = no diagnosis registered during the quarter year). The suspected crime outcome was also modeled with the linear model, but using the count of total number of suspicions registered during a quarter year. All models were adjusted for age (continuous variable), sex, whether the individual was born in Sweden, and group-specific linear time trends. Standard errors were bootstrapped and clustered at the individual level. Models were estimated with the *did_multiplegt*package in Stata, which implements the estimator proposed by de Chaisemartin and d’Haultfoeuille [31]. All confidence intervals were calculated based on 2-sided statistical tests.

We conducted multiple sensitivity analyses, detailed in the Supplement: time-varying treatment exposure; non-linear model specification; addressing potential SUTVA violations; and evaluation of parallel trends assumption violations.

### Within-individual approach

As the second approach, we used a within-individual design to estimate whether sanction type (prison, regular probation, contract treatment) is associated with the risk of outcomes. We took advantage of the fact that a large proportion of people who undergo contract treatment have also served other types of sanctions at some point in time. The individual is used as his or her own control, which automatically adjusts for all time-invariant confounders – even if they are unmeasured. Estimates produced by these models can be interpreted as causal only with the assumption that there is no unaccounted time-varying confounding. We used the same cohort of individuals as in the difference-in-differences approach.

### Follow-up

For each cohort member, we collected all their prison, probation, and contract treatment sanctions served between 1997 and 2013. The dataset was structured in a panel format, where each sanction formed a new observation. For each observation, the follow-up time began at the start of sanction (for prison sanctions, the follow-up started at the start of parole; only sanctions with parole were included). Follow-up ended at outcome, death, emigration from Sweden, administrative censoring 31st December 2013, or by the time a new sanction began, whichever occurred first.

### Measures

Outcomes were defined in the same manner as in difference-in-differences approach. The date of the first diagnosis or crime suspicion after follow-up start was selected for time-to-event modeling. We also included several time-varying covariates. All clients of the Swedish Prison and Probation Service have an implementation plan, which contains rehabilitation measures offered to them during their time as clients. The implementation plan, in turn, is based on assessments made by the staff of the Prison and Probation Service at different points in time. The areas of assessment include alcohol and drug use problems, housing situation, occupation/employment situation, and recidivism risk level. Covariates and their response options are presented in Table S5. For each sanction, we included the assessment registered closest to the start of follow-up. If the closest assessment was registered more than 90 days from the start date, it was excluded. The register has non-uniform coverage for these measures. Alcohol and drug use problems are included since 2006, housing and employment since 2009, and recidivism risk level assessments since 2010.

### Statistical analyses

We used stratified Cox regression which computes a separate hazard function for each individual and estimates whether the discordance in the exposure (type of sanction) is associated with the time-to-event outcome. Covariates were added to the models in a stepwise manner due to the non-uniform register coverage. For each model, only sanctions with start date corresponding to the coverage of the covariates, and with no missing values in any covariate, were included. Specifically, the first model included all sanctions and was adjusted for age and year of sanction start (continuous variables). The second model included sanctions since 2006, and was adjusted for age, year of sanction, alcohol use problems, and drug use problems. The third model included sanctions since 2009, and was adjusted for age, year of sanction, alcohol use problems, drug use problems, housing situation, and occupation/employment situation. The final model included sanctions since 2010, and was adjusted for age, year of sanction, alcohol use problems, drug use problems, housing situation, occupation/employment situation, and the level of recidivism risk. All confidence intervals were calculated based on 2-sided statistical tests.

## Results

**Fig. 1:**
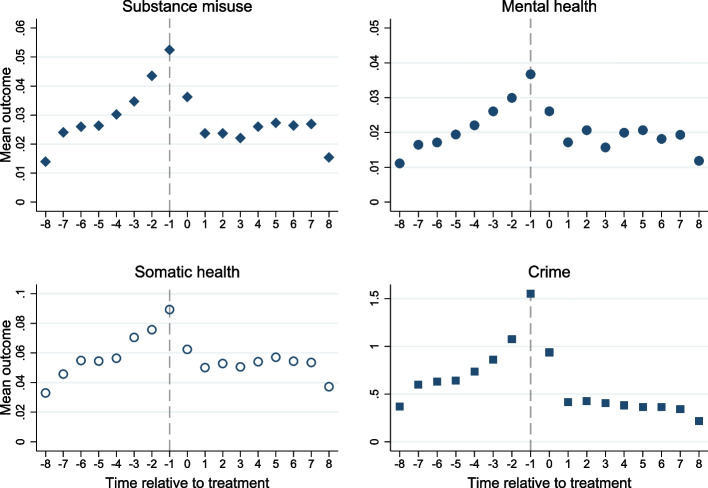
Average outcome levels over the follow-up. Mean outcome level indicates the prevalence of the outcome for the substance misuse, mental health, and somatic health. Mean outcome level indicates the average number of charges/suspicions by the police for the crime outcome. X-axis represents relative time to period (quarter year) where treatment first changes (time 0)

Descriptive characteristics of the cohort (11,893 individuals; 13% women) are presented in Table 1. Figure 1 shows the mean outcome levels over the follow-up, with time structured relative to the quarter year of contract treatment start. All outcomes increased in the pre-treatment period, and then decreased after contract treatment start. Most somatic health outcomes were due to external causes of morbidity, but infections were common as well (Figure S2).

**Table 1:** Descriptive characteristics of the cohort (*n* = 11,893)

	n (%)
Male	10,377 (87.3)
Age (mean, SD)	37.2 (13.1)
Year of sanction (mean, SD)	2006 (3.6)
Born in Sweden	10,178 (85.6)
Education level	
Primary school	5182 (43.6)
High school	5478 (46.1)
University	1029 (8.6)
Unknown	204 (1.7)
Any mental disorder diagnosis	6558 (55.1)
Any substance use disorder diagnosis	5805 (48.8)
Any overdose	1373 (11.5)
Any suicidal behavior	1714 (14.4)
Previous convictions for any crime	
1	608 (5.1)
2–10	4455 (37.5)
> 10	6830 (57.4)
Previous convictions for violent crime	
0	4816 (40.5)
1–5	4852 (40.8)
> 5	2225 (18.7)
Previous imprisonment	3980 (33.5)

Age and education level are based on information recorded in the registers at the time of contract treatment start. Previous diagnoses, convictions, and imprisonments are based on information registered any time by contract treatment start. See Table S1 for definitions and diagnosis codes. Mental health and substance use disorder diagnoses in this table encompass a larger set of ICD codes than the outcome measures (which only include codes for acute conditions). All individuals have at least 1 conviction since receiving contract treatment requires a conviction

### Difference-in-differences

**Fig. 2:**
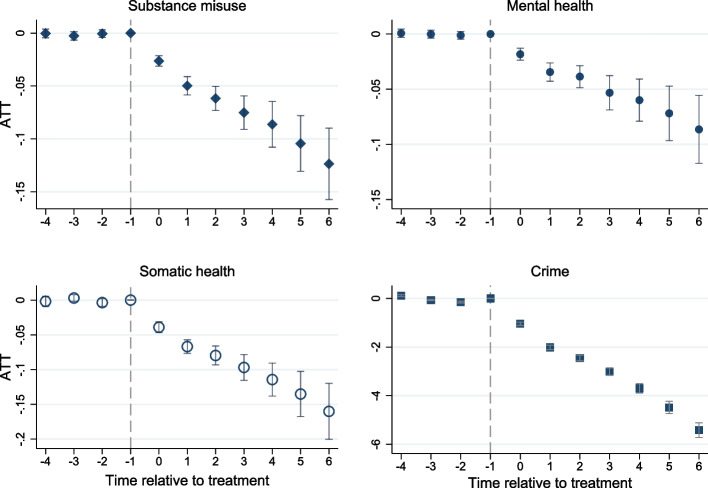
Event study coefficients and their 95% confidence intervals, representing the average treatment effect on the treated (ATT) over the follow-up. ATTs for medical outcomes represent effects in percentage points. ATT for crime represents the effect in the number of charges/suspicions by the police. X-axis represents relative time to period (quarter year) where treatment begins (time 0). Ns used for the estimation of each coefficient are available in Table S6

Figure 2 and Table S11 show event study coefficients from the difference-in-differences models. Pre-treatment trends in the treatment and control groups were indistinguishable from 0. The post-treatment coefficients show that contract treatment reduced all outcomes. The average treatment effect is the weighted average of all post-treatment event study coefficients. The average treatment effect was 7 percentage points for substance misuse, 5 percentage points for mental health, 9 percentage points for somatic health, and 3 fewer charges for crime. The number of observations used in the estimation of event study coefficients is reported in Supplemental Table S6. Sensitivity analyses, which are reported in detail in the Supplemental Results, were consistent with the main results.

### Within-individual analyses

The median number of sanctions in the cohort was 2 (IQR = 1–4), and median number of days between consecutive sanctions was 485 days (IQR: 255–907). A total of 3470 (29%) individuals in the cohort had all 3 types of sanctions between years 1997 and 2013. 5658 (48%) individuals had a prison sanction and a contract treatment sanction, and 4798 (40%) had a regular probation sanction and a contract treatment sanction. Table S7 shows frequencies for the order of different types of sanctions in the cohort. Table 2 shows results from the within-individual Cox regression models comparing times-to-event during periods on probation and contract treatment to periods on parole after prison release. The associations show that the same individual had longer times-to-event for all outcomes during contract treatment than on parole, and this associations persisted even after adjustment for increasingly more time-varying covariates. Table S8 shows results from the same models, but using probation periods as the reference. Associations show longer times-to-event for all outcomes during contract treatment compared to regular probation. However, some of the adjusted associations for substance misuse and mental health outcomes were not statistically significant.

**Table 2:** Within-individual associations of sanction type with substance misuse, mental health, somatic health, and crime

	Outcome, HR (95% CI)			
Model	Substance misuse	Mental health	Somatic health	Crime
**Adjusted for age and year**				
Reference: parole (*n* = 19,864)				
Probation (*n* = 7182)	0.90 (0.82–0.99)	1.06 (0.95–1.18)	0.96 (0.90–1.03)	0.80 (0.77–0.84)
Contract treatment (*n* = 13,508)	0.76 (0.70–0.83)	0.91 (0.82–1.00)	0.85 (0.80–0.90)	0.55 (0.52–0.57)
**Adjusted for age, year, and substance use**				
Reference: parole (*n* = 7945)				
Probation (*n* = 2426)	0.82 (0.69–0.98)	1.01 (0.82–1.26)	0.93 (0.81–1.06)	0.95 (0.87–1.04)
Contract treatment (*n* = 5476)	0.65 (0.56–0.75)	0.78 (0.65–0.94)	0.73 (0.65–0.81)	0.48 (0.44–0.52)
**Adjusted for age, year, substance use, employment, and housing**				
Reference: parole (*n* = 5359)				
Probation (*n* = 1876)	0.80 (0.64–1.00)	0.96 (0.74–1.26)	0.99 (0.83–1.17)	1.05 (0.93–1.18)
Contract treatment (*n* = 4083)	0.70 (0.58–0.86)	0.78 (0.61–0.99)	0.78 (0.68–0.90)	0.50 (0.45–0.55)
**Adjusted for age, year, substance use, employment, housing, and recidivism risk**				
Reference: parole (*n* = 4146)				
Probation (*n* = 1414)	0.86 (0.64–1.15)	0.87 (0.60–1.25)	0.96 (0.76–1.20)	1.16 (0.99–1.35)
Contract treatment (*n* = 2658)	0.64 (0.49–0.85)	0.64 (0.45–0.90)	0.73 (0.59–0.89)	0.45 (0.40–0.51)

The first sensitivity analysis adjusted for time-varying covariate indicating time under supervision, and the estimated remained highly similar to the main results (Table S9). The start of follow-up for prison sanctions was changed to prison entry in the sensitivity analysis (Table S10; start of follow-up was not changed for probation and contract treatment). This changed the results, likely due to direct incapacitation effects of prison. Prison sanctions were associated with longer times-to-event compared to regular probation. In contrast, there was no difference between contract treatment and prison, except for recidivism, where contract treatment was associated with slightly shorter times-to-event.

## Discussion

We found that contract treatment reduced substance misuse, adverse somatic and mental health outcomes, and criminality. It was also associated with the lowest risk of the outcomes compared with time on parole after imprisonment and regular probation. To the best of our knowledge, this is the largest drug court program evaluation to date. We used two quasi-experimental designs, difference-in-differences and a within-individual design, to improve internal validity compared to previous observational studies and to triangulate evidence. These designs rely on different sets of assumptions, and the results should be interpreted with this in mind. We used the staggered rollout design for difference-in-differences, where not-yet-treated individuals were used as controls. This means that pre-treatment time was used as a proxy counterfactual. Most participants spent this time in the community. Estimates from difference-in-differences models should therefore be interpreted as the effect of entering contract treatment as opposed to the “counterfactual” where no intervention was imposed, and individuals continued their life as usual in the community. In contrast, the within-individual design uses the individual as his or her own control. In our setting, individuals’ time on contract treatment was compared to the time they were serving other sanctions. Taken together, our results suggest that contract treatment reduced the studied outcomes compared to the hypothetical alternative of leaving individuals untreated, and was also associated with better outcomes than the alternative sanctions, namely parole and probation.

Our findings for criminality agree with previous meta-analyses of primarily US-based studies [3–5], whereas health effects have been largely overlooked in prior drug court evaluations. We found that contract treatment reduced substance misuse and adverse somatic/mental health outcomes (e.g., overdoses, infections, injuries, mental health emergencies). These results lend support to a meta-analysis, which was statistically underpowered, but found suggestive evidence for reductions in drug use [3]. Only two studies have evaluated other health outcomes for drug court participants. They found no difference between participants and controls in self-reported psychiatric symptoms or physical health [32, 33]. However, both studies had small sample sizes and relied on self-report measures. Differences in results to the current study may therefore be due to statistical power and our outcomes capturing more severe health issues (i.e., emergency health care visits). Overall, our results suggest that besides reducing recidivism, drug courts may also have harm reduction benefits ─ consistent with their ideological aim to approach criminal offending from a public health perspective.

This study may also provide implications concerning implementation of drug courts. The involvement of a judge is a key component of the US drug court system [30]. Drug court participants interact frequently with the judge in the form of status hearings, where the judge gives out praise or sanctions, depending on participants’ progress and conduct. The Swedish implementation contains no judge oversight or sanctions, suggesting that these components are not necessary for an effective intervention. While our study cannot establish whether outcomes would have been even better had judges been involved, emerging evidence from the US shows that more frequent status hearings and use of sanctions do not necessarily improve effectiveness [3, 4, 11]. Further, our results suggest that a program with less strict eligibility criteria than what is typical in the US can be effective. Although programs with similarly inclusive eligibility requirements are rare in the US, there is suggestive evidence that expanding the availability of drug courts might prove successful in the US context as well [19]. It may be possible to reform frequently criticized features of US drug court system [13–18] while maintaining program’s effectiveness.

### Strengths and limitations

This study has several limitations. First, since the outcomes were based on emergency inpatient and outpatient health care visits and police records, they do not capture events such as health problems treated in primary care or criminality undetected by the police. Second, the causal interpretation of treatment effects rests on assumptions. For difference-in-differences, the key assumption is the parallel trends assumption. The robust inference sensitivity analysis indicated that the early treatment effects were robust to large non-linear violations to the parallel trends assumption, whereas causal interpretation for the later treatment effects requires that non-linear violations are not very large. It seems unlikely that this group with very poor physical and psychosocial functioning would have experienced an extreme, spontaneous improvement in the absence of any intervention – which is what a large non-linear violation to the parallel trends would imply. For within-individual analyses, the assumption is that there is no unadjusted time-varying confounding. While we were able to adjust for many important time-varying covariates, the possibility of residual confounding cannot be entirely ruled out. Further, the availability of the covariates was limited to certain years, which reduced sample size when more covariates were added to the model. Nevertheless, effect sizes were highly consistent regardless of sample composition. A further caveat relating to the within-individual analyses concerns the start of follow-up. In the main analyses, follow-up for prison sanctions started at the time of parole, since we assumed that parole would be comparable to probation and contract treatment in terms of supervision and setting. However, contract treatment was no longer superior to prison when start of follow-up was changed to prison entry for prison sanctions. This shows that, as expected, prison has major incapacitation effects, but contract treatment nevertheless produced comparable times-to-event for all outcomes but recidivism, where times-to-event were slightly shorter.

## Conclusions

To conclude, this study shows that the Swedish version of drug court, the contract treatment sanction, reduces criminality and substance-related heath harms. Importantly, it was more effective than imprisonment and probation without a treatment component. These findings contain several clinical and policy implications. First, contract treatment serves multiple purposes by reducing outcomes relevant for public health as well as the criminal justice system. Second, our finding of reduced substance-related health harms suggests that drug courts could be an important policy tool for reducing health inequalities. Third, the US drug court system has been criticized for having punitive features (e.g., use of jailtime as punishment for technical violations) and strict eligibility criteria that exclude people with the greatest need for treatment. Our study suggests that an implementation approach that is less punitive and more inclusive than what is typical in the US can be successful. Thus, it may be possible to reform frequently criticized features of US drug court system while maintaining program’s effectiveness. Future studies could expand our findings in several ways: for example, by investigating a similar set of outcomes in other contexts (such as in the US), specifying which elements of the intervention are effective and for whom, and by clarifying how implementation strategies affect program effectiveness (e.g., eligibility criteria, use of sanctions for technical violations).

### Supplementary Information

**Supplementary Material 1. **

## Data Availability

Swedish legislation prevents sharing of sensitive or confidential data. According to the Swedish Ethical Review Act, the Personal Data Act, and the Administrative Procedure Act, data can only be made available, after ethical and legal review, for researchers who meet the criteria for access to this type of sensitive and confidential data. Researchers interested in obtaining Swedish register data can consult Swedish Research Council’s website for register-based research (https://www.registerforskning.se/en/).
