# Supplementary material for: Effectiveness of substance use disorder treatment as an alternative to imprisonment
Source: BMC Psychiatry. 2024 Apr 9;24:260. doi: 10.1186/s12888-024-05734-y (PMC11003076; doi:10.1186/s12888-024-05734-y)
Supplement: Supplementary file 1 — Supplementary Material 1. [file 12888_2024_5734_MOESM1_ESM.docx]

**Supplemental Materials**

**Supplemental Methods**

**Supplemental Results**

**Figure S1. Illustration of the data structure in difference-in-differences analysis**

**Table S1. Data sources and definitions for cohort characteristics**

**Table S2. ICD-10 codes for defining acute substance misuse events**

**Table S3. ICD-10 codes for defining acute mental health events**

**Table S4. ICD-10 codes for defining acute somatic health events**

**Table S5. Covariates for the within-individual analysis**

**Figure S2. Prevalence of somatic diagnoses over the pre-treatment time**

**Table S6. Number of observations used in the estimation of event study coefficients in Manuscript Figure 2**

**Table S7. Frequencies for the order of different types of sanctions in the cohort**

**Table S8. Within-individual Cox regression models estimating associations of sanction type with substance misuse, mental health, somatic health, and crime (probation as reference)**

**Table S9. Within-individual Cox regression models estimating associations of sanction type with substance misuse, mental health, somatic health, and crime (controlling for time-varying indicator for time under supervision)**

**Table S10. Within-individual Cox regression models estimating associations of sanction type with substance misuse, mental health, somatic health, and crime (start of follow-up at entry to prison for prison sanctions)**

**Figure S3. Difference-in-differences sensitivity analysis with time-varying treatment**

**Table S11. Comparison of difference-in-differences treatment effects from the linear model with logit and Poisson models**

**Figure S4. Difference-in-differences sensitivity analysis with cohort restricted to sanctions starting in 2003 and individuals without any prison or probation time in the pre-treatment period.**

**Figure S5. Robust inference sensitivity analysis for substance misuse**

**Figure S6. Robust inference sensitivity analysis for mental health**

**Figure S7. Robust inference sensitivity analysis for somatic health**

**Figure S8. Robust inference sensitivity analysis for crime**

**Supplemental Methods**

Contract treatment details and institutional context

Substance misuse-related cases do not have a separate court docket but are handled in the same manner as any other court case. If the committed crime would result in a prison sentence of 2 years or less and the judge has a reason to believe that the committed crime was closely linked to defendant’s substance use problem, the defendant becomes eligible for *kontraktsvård* (directly translated as contract treatment). This means that cases involving violent crimes and more serious drug-related crimes are not automatically excluded from eligibility. Contract treatment is a version of probation that involves undergoing substance use disorder treatment combined with supervision by a probation officer. The convicted person consents to treatment, and by doing so, avoids a sentence in prison. Treatment is offered by health and social care providers in the community and tailored to the specific needs of the individual. It may entail psychological and/or pharmacological treatment delivered in an inpatient or outpatient setting. Treatment length varies between 1 month and up to 2 years. Supervision by a probation officer typically lasts for at least 1 year. Participation is entirely cost-free for the participant. If the client drops out of treatment prematurely, it is up to the court’s discretion to decide whether the remaining part of the sentence must be served in prison. The judge ultimately decides whether the sanction is given or not but does not have power to regulate or intervene in the actual treatment process in any way. The client is not subjected to court appearances where his or her treatment progress is praised or castigated. Registers do not contain information on whether treatment was completed or not, and thus all analyses included treatment initiators (as in an intention-to-treat design).

Sensitivity analyses: difference-in-differences approach

*Time-varying treatment.* The staggered rollout design considers treatment status to be permanent, i.e., once individual becomes treated, they remain treated for the rest of the panel. However, this might not be accurate in the context of this study, as contract treatment involves treatment in combination with supervision by a probation officer, both of which will end eventually. The Prison and Probation Service’s register does not contain information on time spent in treatment, but has records for time spent under supervision since 2003. For a subset of cohort whose sanction started in 2003 or later, 89% were no longer under supervision by the end of follow-up. To account for this, we conducted a sensitivity analysis where treatment was considered time-varying. At the start of follow-up, treatment indicator was coded as 0. The indicator switched to 1 on the quarter year sanction began, and switched back to 0 on the first quarter year after supervision period had ended.

*Non-linear difference-in-differences.* Although the linear probability model is a standard estimator in many fields, there may still be concerns that it is inappropriate for binary outcomes. The main concern is that the model can make predictions that are outside of bounds for the outcome. Similarly, the crime outcome is a count variable, where the Poisson model may be more appropriate than the linear model. To check whether the linear models may have been misspecified, we estimated the models again with a non-linear difference-in-differences estimator proposed by Wooldrigde [1], which can fit logit and Poisson models. We computed marginal effects from the fitted models, which can be directly compared with the estimates obtained from the linear models. Analyses were conducted with the *etwfe* package in R.

*Addressing potential SUTVA violations.* SUTVA contains the assumption that several versions of treatment do not exist. Contract treatment violates this assumption, as individuals receive treatment that is tailored to their individual needs. Hernán and Vanderweele show that ATT is identifiable even with several versions of treatment, as long as there is only 1 version of the control [2]. The control condition in our setting is the pre-treatment time. Most individuals spent this time in the community. However, 23% spent at least some time in prison or under regular probation. We conducted a sensitivity analysis where these individuals were removed from the cohort to homogenize the control condition.

*The robust inference framework.* The credibility of difference-in-differences design rests on the validity of the parallel trends assumption: in the absence of treatment, the outcomes in treatment and control groups would have continued to develop in parallel. While the pre-treatment trends can be evaluated empirically, which provides some support for this assumption, the counterfactual post-treatment trend in the treatment group is always unobserved. All outcomes tended to increase over the pre-treatment period, which suggests that regression to the mean might be a concern in the context of this study. Perhaps the 2-year period preceding contract treatment was a particularly bad time in the individuals’ life, and outcomes would have been reduced to some degree even without the intervention. The “true” counterfactual post-treatment trend might slope downwards, while the observed trend in controls was increasing linearly (because they were still in their pre-treatment period). This produces biased estimates, as the assumption is that the counterfactual of the treatment group follows the observed trend of the controls. A sensitivity analysis proposed by Rambachan and Roth can be used to calculate a “breakdown value”, i.e., how severe the parallel trends violation would have to be so that the original treatment effects would no longer be significant [3]. We used their smoothness restriction approach. When imposing a smoothness restriction, the assumption is that the underlying trend does not deviate *too much* from linearity. Specifically, the change in the slope of the underlying trend (the second difference in δ) be no more than *M* between consecutive periods, where *M* is a parameter specified by the researcher. We selected 0.02 as the maximum value of *M* for the medical outcomes. This means that the differential trends change by at most 0.02 units in absolute value (2 percentage points) between consecutive periods. Given our particular effect scaling, this would allow for substantial non-linearity in the underlying trend. We chose 1 as the maximum value of *M* for the crime outcome, which has a different scaling to the other outcomes. Given this scaling, *M* of 1 allows for substantial non-linearity in the underlying trend. The sensitivity analysis was applied to event study coefficients at t0, t2, and t4. Analyses were conducted with the *HonestDiD* package in Stata.

Sensitivity analyses: within-individual approach

First, we tested whether time spent under supervision during follow-up influenced the results. For this analysis, we selected all sanctions since 2003 with confirmed supervision time. Follow-up time was split based on the date when supervision period ended. The final models were adjusted for time-varying covariate indicating time under supervision vs. not under supervision. The analysis ensures that follow-up time always includes supervision from Prison and Probation Service, making these different types of sentences more comparable, while the time-varying covariate accounts for potentially different lengths of supervision period in different sanction types.

The start of follow-up for prison sanctions was defined as the start of parole in the main analysis. This makes the follow-up time as comparable as possible between each type of sanction, as then all of them take place in the community and contain supervision by the Prison and Probation Service. However, the actual treatment sequence for prison sanctions starts at entry to prison. While in prison, individuals have a significantly reduced probability of experiencing the outcomes due to incapacitation. Incapacitation effects are much larger than effects of supervision in the community setting, as prisoners are actively prevented from certain behaviors, such as committing crimes, using substances, or harming themselves. Further, low-penalty crimes committed in prison may sometimes be recorded as disciplinary infractions, and not reported to the police. But since none of the outcomes are impossible even during incarceration, this allows us to further investigate the role of incapacitation. For this analysis, we changed the start of follow-up for prison sanctions to prison entry. Assuming prison has larger incapacitation effects at the start of follow-up than do probation and contract treatment, this should produce longer times-to-event for the prison sanctions.

**References**

1. Wooldridge, J. (2023). Simple approaches to nonlinear difference-in-differences with panel data. The Econometrics Journal, 26(3), C31–C66.
2. VanderWeele, T. J., & Hernan, M. A. (2013). Causal inference under multiple versions of treatment. Journal of Causal Inference, 1(1), 1-20.
3. Rambachan, A., & Roth, J. (2023). A more credible approach to parallel trends. Review of Economic Studies, rdad018.

**Supplemental Results**

Sensitivity analyses: difference-in-differences approach

In the first sensitivity analysis where treatment status was time-varying, the median length of treatment was 365 days (IQR: 365-396). The event study coefficients remained very similar to the original results (Figure S3).

In the second sensitivity analysis using non-linear models (Table S11), the logit model estimates for the medical outcomes were highly consistent with the original linear model coefficients. Poisson model estimates for the crime outcome were slightly lower than the linear model estimates.

In the third sensitivity analysis, excluding individuals who had been in prison or under probation during pre-treatment did not change the results (Figure S4).

In the main analysis, we observed that pre-treatment trends in the treatment and control groups were indistinguishable from 0, except for crime, where pre-trends showed very minor deviations. Rambachan and Roth argue that, for several reasons, this traditional approach of using null hypothesis testing for pre-trends should be avoided [1-2]. One of their reasons is that outcome trends are never exactly parallel, and a more relevant issue is whether the potential violations induce meaningful bias to the treatment effects [1]. This is why we applied the robust inference sensitivity analysis to our results, rather than discarded the analysis entirely because of this pre-trend finding. Figures S5 to S8 show robust confidence intervals of event study coefficients at t0, t2, and t4 calculated for different values of M (parameter for non-linear divergence in the underlying trends). As expected, the latter event study coefficients were much more sensitive to non-linear violations of the parallel trends assumption than the earlier coefficients. This is due to the accumulation of bias over consecutive periods. The analyses suggest that treatment effects in the early periods of treatment were robust to large non-linear violations of the parallel trends assumption. In contrast, assumption of no large non-linear violations to the parallel trends is required to interpret the latter treatment effects as causal.

**References**

1. Rambachan, A., & Roth, J. (2023). A more credible approach to parallel trends. *Review of Economic Studies*, rdad018.
2. Roth, J. (2022). Pretest with caution: Event-study estimates after testing for parallel trends. *American Economic Review: Insights*, 4(3), 305-322


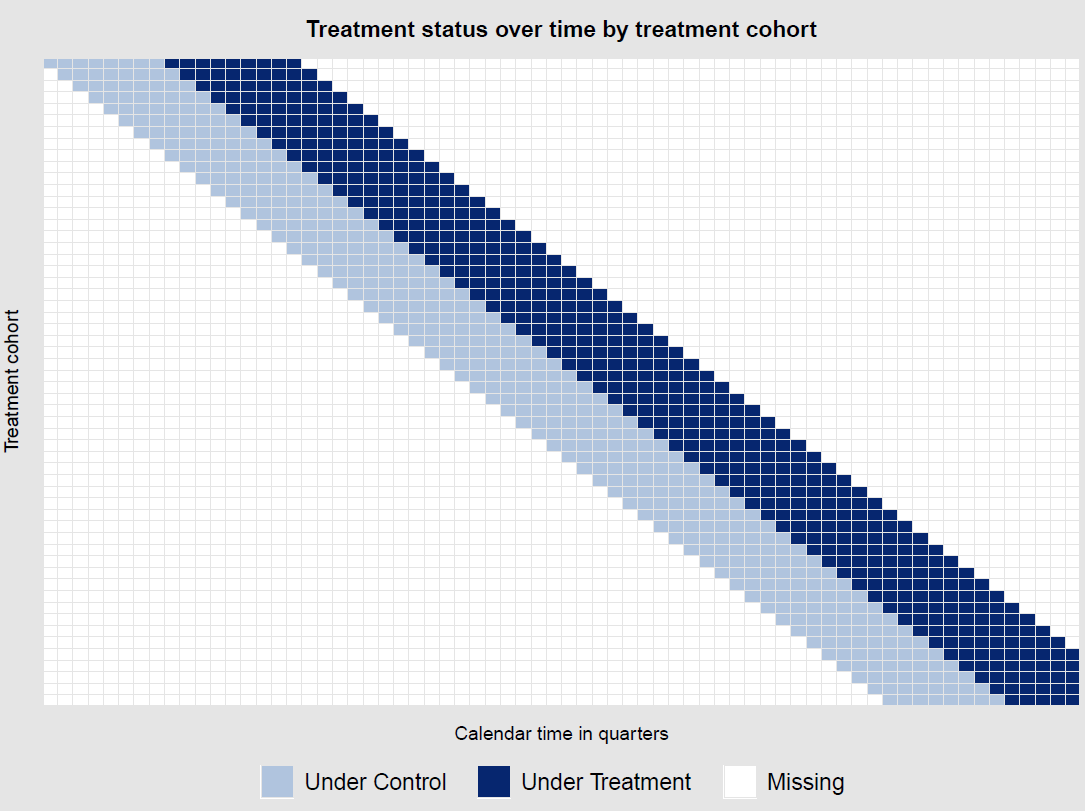


Figure S1. Illustration of the data structure in difference-in-differences analysis. Each square represents a quarter year. X-axis represents calendar time in quarters (the first quarter is Q1 1997, the last is Q4 2013). Y-axis represents a “treatment cohort” that becomes treated at the same quarter year (the first treatment cohort starts at Q1 1999, the last at Q4 2012).

|  | **Source of data/name of register** | **Timing of measurement** | **ICD codes** |
| --- | --- | --- | --- |
| Sex | Total population register | N/A | N/A |
| Age | Total population register | At contract treatment start | N/A |
| Year of sanction | Prison and probation services register | At contract treatment start | N/A |
| Birth country | Total population register | N/A | N/A |
| Education | The longitudinal integrated database for health insurance and labour market studies | Highest level achieved by contract treatment start | N/A |
| Any mental disorder diagnosis | National patient register | Registered any time by contract treatment start | F00-F99 |
| Any substance use disorder diagnosis | National patient register | Registered any time by contract treatment start | F10-F19 |
| Any overdose | National patient register | Registered any time by contract treatment start | X40-X49, T36-F51 |
| Any suicidal behavior | National patient register | Registered any time by contract treatment start | X60-X84, Y10-Y34 |
| Number of convictions for any crime | Swedish national council for crime prevention register | Registered any time by contract treatment start | N/A |
| Previous convictions for violent crime | Swedish national council for crime prevention register | Registered any time by contract treatment start | N/A |
| Previous imprisonment | Prison and probation services register | Registered any time by contract treatment start | N/A |

Table S1. Data sources and definitions for cohort characteristics

**Table S2.** ICD-10 codes for defining acute substance misuse events

| Name | ICD-10 code |
| --- | --- |
| Acute intoxication | F10.0-F16.0, F18.0-F19.0 |
| Withdrawal state | F10.3-F16.3, F18.3-F19.3 |
| Withdrawal state with delirium | F10.4-F16.4, F18.4-F19.4 |
| Substance-induced psychosis | F10.5-F16.5, F18.5-F19.5 |
| Poisoning by drugs or alcohol | T36-T51, T65 |
| Accidental poisoning by drugs or alcohol | X40-X45, X49, |
| Poisoning by drugs or alcohol (undetermined intent) | Y10-Y15 |
| Evidence of alcohol involvement | Y91 |

Table S3. ICD-10 codes for defining acute mental health events

| Name | ICD-10 code |
| --- | --- |
| Acute psychotic episode | F23, F29 |
| Acute manic episode | F30, F31.0, F31.1, F31.2 |
| Depressive episode | F31.3-F31.6, F32, F38, F39 |
| Anxiety and stress disorder | F40, F41, F43 |
| Self-harm and suicidality | X60-X84, Y20-Y34 |

**Table S4.** ICD-10 codes for defining acute somatic health events

| Name | ICD-10 code |
| --- | --- |
| Infectious and parasitic diseases | A00-B99 |
| Conjunctivitis | H10 |
| Circulatory system disease |  |
| Acute rheumatic fever | I00-I02 |
| Essential (primary) hypertension | I10 |
| Angina pectoris | I20 |
| Acute myocardial infarction | I21 |
| Subsequent myocardial infarction | I22 |
| Complications following acute myocardial infarction | I23 |
| Other acute ischaemic heart diseases | I24 |
| Pulmonary embolism | I26 |
| Acute pericarditis | I30 |
| Acute and subacute endocarditis | I33 |
| Cardiac arrest | I46 |
| Atrial fibrillation and flutter | I48 |
| Other cardiac arrhythmias | I49 |
| Haemorrhage | I60-I62 |
| Infarction | I63 |
| Stroke | I64 |
| Aortic aneurysm and dissection | I71 |
| Respiratory system disease |  |
| Acute upper respiratory infections | J00-J06 |
| Influenza and pneumonia | J09-J18 |
| Other acute lower respiratory infections | J20-J22 |
| Other diseases of upper respiratory tract | J30-J39 |
| Digestive system disease |  |
| Ulcer | K25-K28 (fourth digit 0-3) |
| Gastritis and duodenitis | K29 (fourth digit 0-3) |
| Appendicitis | K35-K37 |
| Alcoholic hepatic failure | K70.4 |
| Toxic liver disease | K71 |
| Acute and subacute hepatic failure | K72.0 |
| Skin disease |  |
| Infections of the skin | L00-L08 |
| Dermatitis due to substances taken internally | L27 |
| Infectious arthropathies | M00-M03 |
| Genitourinary system disease |  |
| Acute tubulo-interstitial nephritis | N10 |
| Calculus of kidney, ureter, and lower urinary tract | N20-N21 |
| Cystitis | N30 |
| Cough, breathing abnormalities | R05-R06 |
| External causes of morbidity |  |
| Injuries | S00-S99, T00-T35, T66-T78 |
| Transport accidents | V01-V99 |
| Other external causes of accidental injury | W00-X39, |
| Assault | X85-Y09 |

**Table S5.** Covariates for the within-individual analysis

| **Covariate** | **Response options** |
| --- | --- |
| Alcohol use | No problems |
|  | Alcohol misuse. Exists if the client has drunk alcohol to such an extent that it has caused physical, psychological or social harm to him/her or those close to him/her during the past 12 months. |
| Drug use | No problems |
|  | Drug misuse. Exists if the client has used drugs during the last twelve months, but does not fall under the definition of serious drug abuse. |
|  | Serious drug addiction. Exists if a person has injected drugs one or more times during the last twelve months or used drugs in another way (for example, smoked hashish) daily or almost daily in the last twelve months. |
| Housing | Homeless |
|  | Stable housing. Lives in supported housing (e.g., with parents, in institution, halfway-house) |
|  | Stable housing. Lives independently. |
| Employment | Unemployed |
|  | On sick leave |
|  | Placed in substance use disorder treatment |
|  | Outside of work force (e.g., retirement, maternity leave) |
|  | Participates in supported employment program, work trial etc. |
|  | Employed or in education |
| Recidivism risk | Low |
|  | Medium |
|  | High |


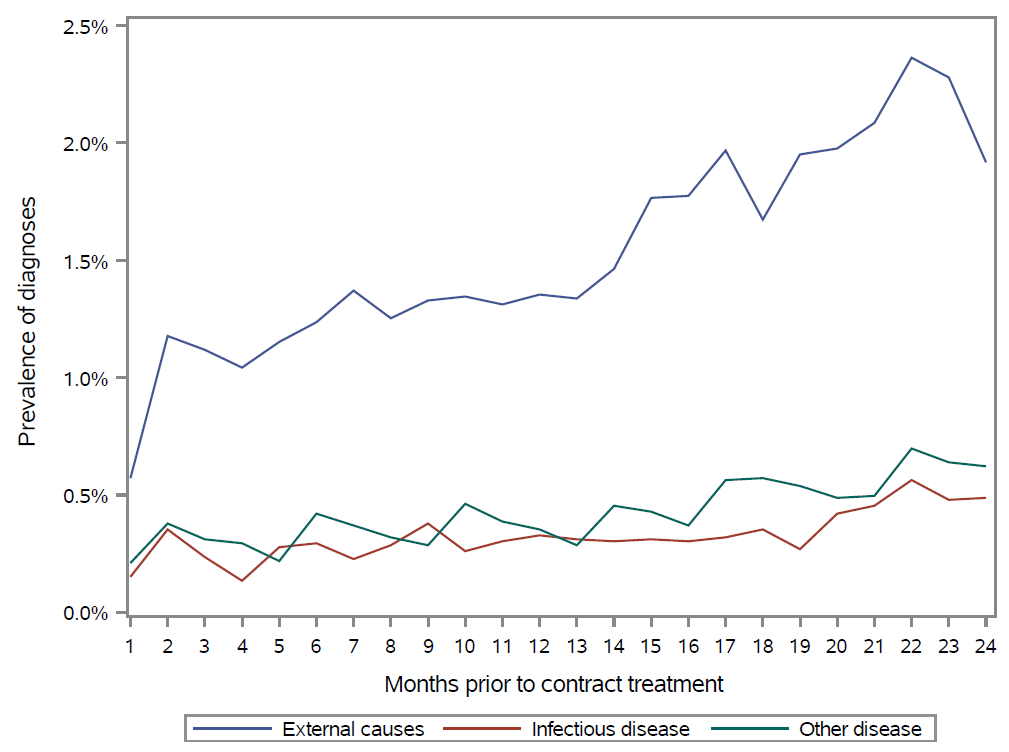


**Figure S2. Prevalence of somatic diagnoses over the pre-treatment time.** Month 24 is the last month before the start of contract treatment.

**Table S6.** Number of observations used in the estimation of event study coefficients in Manuscript Figure 2

|  | **Substance misuse** | | **Mental health** | | **Somatic health** | | **Crime** | |
| --- | --- | --- | --- | --- | --- | --- | --- | --- |
|  | n | Switchers | n | Switchers | n | Switchers | n | Switchers |
| t-4 | 33709 | 10787 | 33709 | 10787 | 33709 | 10787 | 33709 | 10787 |
| t-3 | 57404 | 11445 | 57404 | 11445 | 57404 | 11445 | 57404 | 11445 |
| t-2 | 80657 | 11675 | 80657 | 11675 | 80657 | 11675 | 80657 | 11675 |
| t0 | 91818 | 11675 | 91818 | 11675 | 91818 | 11675 | 91818 | 11675 |
| t1 | 79810 | 11445 | 79810 | 11445 | 79810 | 11445 | 79810 | 11445 |
| t2 | 67479 | 10787 | 67479 | 10787 | 67479 | 10787 | 67479 | 10787 |
| t3 | 55207 | 10113 | 55207 | 10113 | 55207 | 10113 | 55207 | 10113 |
| t4 | 43027 | 9422 | 43027 | 9422 | 43027 | 9422 | 43027 | 9422 |
| t5 | 31146 | 8900 | 31146 | 8900 | 31146 | 8900 | 31146 | 8900 |
| t6 | 19411 | 8408 | 19411 | 8408 | 19411 | 8408 | 19411 | 8408 |
| Avg. ATT | 387898 | 70750 | 387898 | 70750 | 387898 | 70750 | 387898 | 70750 |

**Table S7**. Frequencies for the order of different types of sanctions in the cohort

|  | |  |  |
| --- | --- | --- | --- |
| Individuals with 3 types of sanction | Type of sanction | |  |
| Order of sanction | Prison | Probation | Contract treatment |
| First | 991 (28.6%) | 1954 (56.3%) | 525 (15.1%) |
| Second | 2055 (59.2%) | 714 (20.6%) | 701 (20.2%) |
| Third | 2051 (59.1%) | 654 (18.9%) | 765 (22.1%) |
| Individuals with at least 2 types of sanction | | |  |
| First | 2137 (30.6%) | 2804 (40.2%) | 2042 (29.2%) |
| Second | 3626 (51.9%) | 1294 (18.5%) | 2063 (29.6%) |
| Third | 3014 (59.0%) | 838 (16.4%) | 1260 (24.7%) |

**Table S8.** Within-individual Cox regression models estimating associations of sanction type with substance misuse, mental health, somatic health, and crime (probation as the reference)

|  | Outcome, HR (95% CI) |  |  |  |
| --- | --- | --- | --- | --- |
| Model | Substance misuse | Mental health | Somatic health | Crime |
| **Adjusted for age and year** |  |  |  |  |
| Reference: probation (n=7182) |  |  |  |  |
| Parole (n=19864) | 1.11 (1.01-1.22) | 0.95 (0.85-1.06) | 1.04 (0.98-1.11) | 1.25 (1.19-1.30) |
| Contract treatment (n=13508) | 0.84 (0.77-0.93) | 0.86 (0.77-0.96) | 0.88 (0.83-0.95) | 0.69 (0.66-0.72) |
| **Adjusted for age, year, and substance use** | |  |  |  |
| Reference: probation (n=2426) |  |  |  |  |
| Parole (n=7945) | 1.22 (1.02-1.45) | 0.98 (0.79-1.22) | 1.08 (0.95-1.24) | 1.05 (0.96-1.15) |
| Contract treatment (n=5476) | 0.79 (0.65-0.96) | 0.77 (0.61-0.98) | 0.79 (0.68-0.91) | 0.50 (0.45-0.56) |
| **Adjusted for age, year, substance use, employment, and housing** | | |  |  |
| Reference: probation (n=1876) |  |  |  |  |
| Parole (n=5359) | 1.25 (0.99-1.57) | 1.04 (0.79-1.36) | 1.01 (0.85-1.21) | 0.95 (0.85-1.07) |
| Contract treatment (n=4083) | 0.88 (0.68-1.14) | 0.80 (0.59-1.10) | 0.79 (0.65-0.96) | 0.47 (0.42-0.54) |
| **Adjusted for age, year, substance use, employment, housing, and recidivism risk** | | | |  |
| Reference: probation (n=1414) |  |  |  |  |
| Parole (n=4146) | 1.16 (0.87-1.56) | 1.15 (0.80-1.66) | 1.05 (0.84-1.31) | 0.87 (0.74-1.00) |
| Contract treatment (n=2658) | 0.75 (0.53-1.05) | 0.74 (0.46-1.17) | 0.76 (0.58-0.99) | 0.39 (0.33-0.47) |

**Table S9.** Within-individual Cox regression models estimating associations of sanction type with substance misuse, mental health, somatic health, and crime (controlling for time-varying indicator for time under supervision)

|  | Outcome, HR (95% CI) |  |  |  |
| --- | --- | --- | --- | --- |
| Model | Substance misuse | Mental health | Somatic health | Crime |
| **Adjusted for age, year, and supervision** |  |  |  |  |
| Reference: parole (n=5523) |  |  |  |  |
| Probation (n=2884) | 0.83 (0.71-0.98) | 1.00 (0.83-1.20) | 0.96 (0.86-1.08) | 0.90 (0.82-0.98) |
| Contract treatment (n=8748) | 0.74 (0.64-0.86) | 0.88 (0.74-1.04) | 0.82 (0.74-0.90) | 0.60 (0.56-0.65) |

**Table S10.** Within-individual Cox regression models estimating associations of sanction type with substance misuse, mental health, somatic health, and crime (start of follow-up at entry to prison for prison sanctions)

|  | Outcome,  HR (95% CI) |  |  |  |
| --- | --- | --- | --- | --- |
| Model | Substance misuse | Mental health | Somatic health | Crime |
| **Adjusted for age and year** |  |  |  |  |
| Reference: prison (n=22311) |  |  |  |  |
| Probation (n=7191) | 1.22 (1.11-1.34) | 1.23 (1.11-1.37) | 1.23 (1.16-1.32) | 1.76 (1.68-1.84) |
| Contract treatment (n=13518) | 1.03 (0.94-1.11) | 1.06 (0.96-1.17) | 1.08 (1.02-1.14) | 1.14 (1.09-1.18) |
| **Adjusted for age, year, and substance use** | |  |  |  |
| Reference: prison (n=7932) |  |  |  |  |
| Probation (n=2432) | 1.25 (1.04-1.50) | 1.22 (0.98-1.51) | 1.37 (1.20-1.56) | 2.35 (2.12-2.60) |
| Contract treatment (n=5479) | 0.99 (0.85-1.15) | 0.96 (0.80-1.14) | 1.05 (0.94-1.18) | 1.22 (1.13-1.31) |
| **Adjusted for age, year, substance use, employment, and housing** | | |  |  |
| Reference: prison (n=5663) |  |  |  |  |
| Probation (n=1882) | 1.27 (1.00-1.61) | 1.31 (0.98-0.73) | 1.36 (1.16-1.61) | 2.69 (2.36-3.07) |
| Contract treatment (n=4084) | 1.08 (0.89-1.31) | 1.05 (0.83-1.33) | 1.07 (0.92-1.24) | 1.35 (1.23-1.49) |
| **Adjusted for age, year, substance use, employment, housing, and recidivism risk** | | | |  |
| Reference: prison (n=4319) |  |  |  |  |
| Probation (n=1417) | 1.56 (1.15-2.12) | 1.17 (0.80-1.70) | 1.32 (1.06-1.65) | 2.79 (2.36-3.01) |
| Contract treatment (n=2660) | 1.07 (0.83-1.39) | 1.00 (0.73-1.37) | 1.08 (0.89-1.32) | 1.30 (1.14-1.48) |


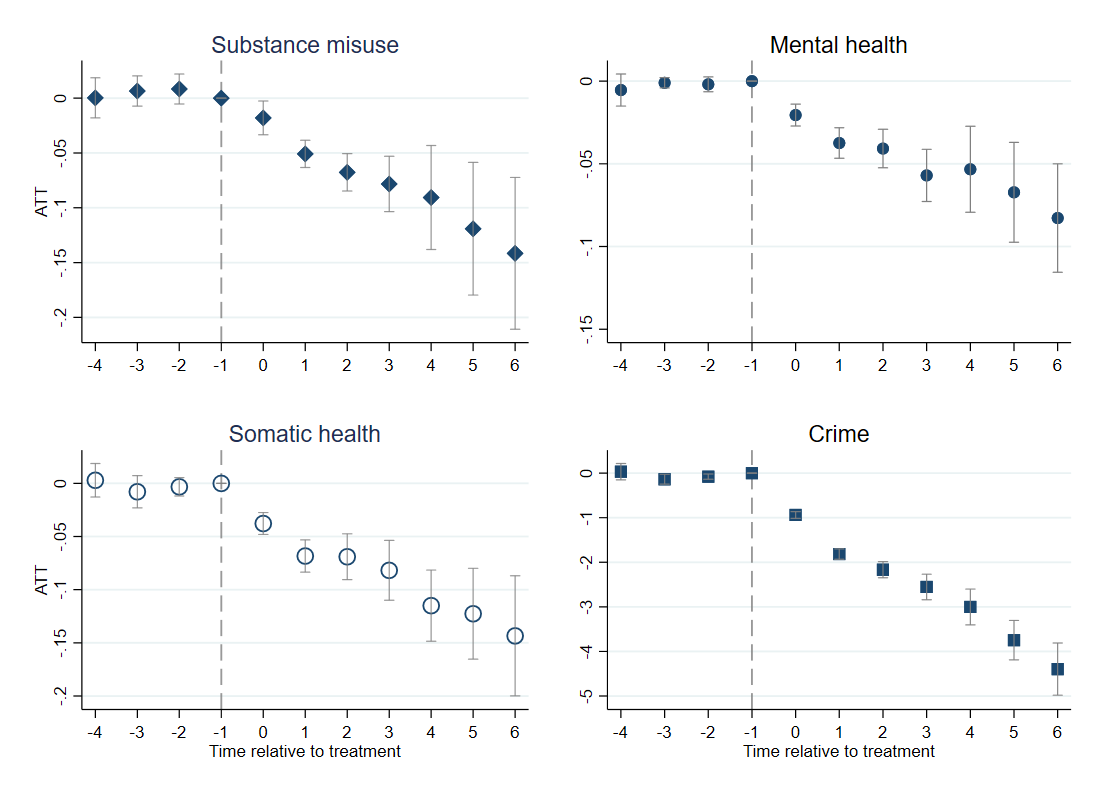


Figure S3. Difference-in-differences sensitivity analysis with time-varying treatment. Figure shows event study coefficients and their 95% confidence intervals, representing the average treatment effect on the treated (ATT) over the follow-up. ATTs for medical outcomes represent effects in percentage points. ATT for crime represents the effect in the number of charges/suspicions by the police. X-axis represents relative time to period (quarter year) where treatment first changes (time 0).

**Table S11.** Comparison of difference-in-differences treatment effects from the linear model with logit and Poisson models

| Outcome | t0 | t1 | t2 | t3 | t4 | t5 | t6 |
| --- | --- | --- | --- | --- | --- | --- | --- |
| Substance misuse |  |  |  |  |  |  |  |
| Linear | -.026 (-.031, -.021) | -.050 (-.058, -.041) | -.062 (-.072, -.050) | -.075 (-.091, -.059) | -.086 (-.108, -.065) | -.104 (-.131, -.078) | -.124 (-.157, -.090) |
| Logit | -.024 (-.028, -.020) | -.047 (-.051, -.044) | -.060 (-.063, -.056) | -.074 (-.078, -.071) | -.085 (-.090, -.081) | -.103 (-.108, -.096) | -.122 (-.128, -.117) |
| Mental health |  |  |  |  |  |  |  |
| Linear | -.018 (-.021, -.013) | -.034 (-.042, -.026) | -.039 (-.049, -.029) | -.053 (-.069, -.038) | -.060 (-.079, -.041) | -.072 (-.097, -.047) | -.086 (-.117, -.056) |
| Logit | -.017 (-.021, -.014) | -.033 (-.036, -.030) | -.037 (-.041, -.034) | -.053 (-.056, -.050) | -.060 (-.064, -.056) | -.071 (-.075, -.066) | -.087 (-.091, -.082) |
| Somatic health |  |  |  |  |  |  |  |
| Linear | -.039 (-.047, -.031) | -.067 (-.077, -.057) | -.080 (-.093, -.066) | -.097 (-.115, -.078) | -.114, (-.138, -.091) | -.135, (-.168, -.103) | -.160 (-.200, -.120) |
| Logit | -.037 (-.043, -.032) | -.066 (-.071, -.061) | -.080 (-.085, -.074) | -.096 (-.102, -.090) | -.109 (-.116, -.103) | -.130 (-.137, -.122) | -.155 (-.162, -.147) |
| Crime |  |  |  |  |  |  |  |
| Linear | -1.04 (-1.11, -.974) | -2.01 (-2.10, -1.93) | -2.45 (-2.57, -2.33) | -3.01 (-3.17, -2.86) | -3.70 (-3.89, -3.50) | -4.49 (-4.74, -2.24) | -5.42 (-5.72, -5.11) |
| Poisson | -.753 (-.788, -.719) | -1.63 (-1.65, -1.60) | -2.03 (-2.06, -2.00) | -2.55 (-2.58, -2.52) | -3.18 (-3.21, -3.15) | -3.95 (-3.98, -3.92) | -4.86 (-4.89, -4.83) |


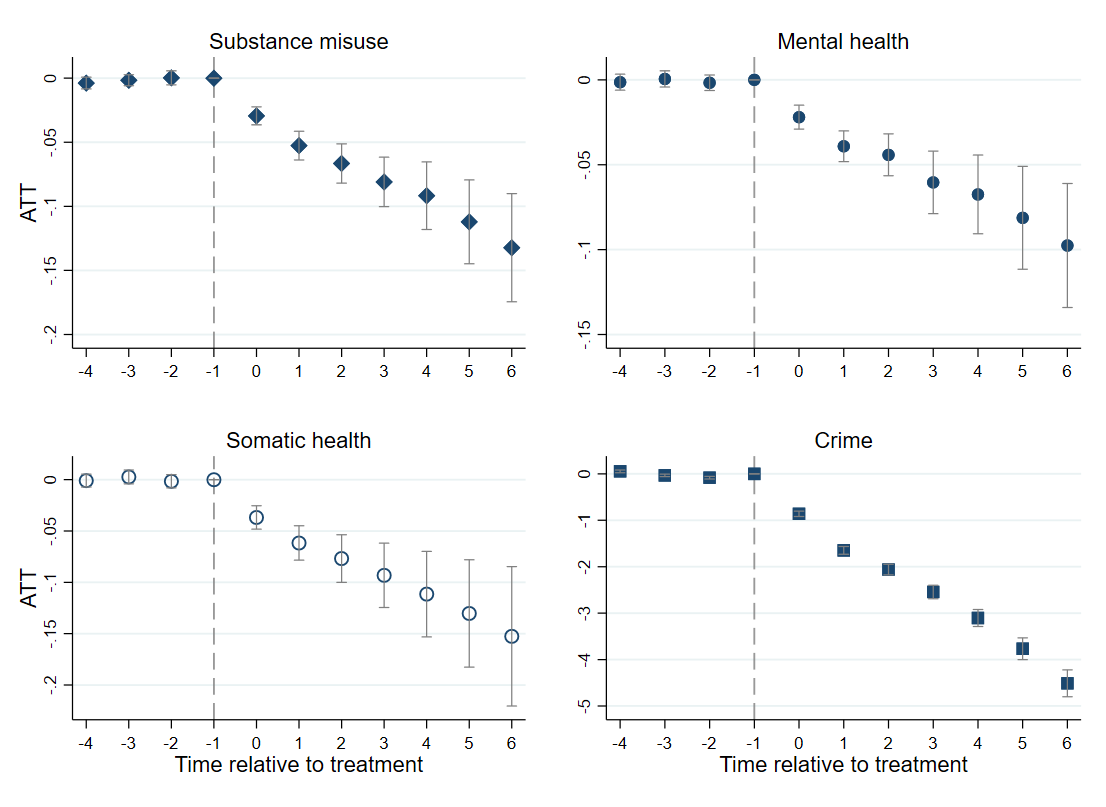


**Figure S4. Difference-in-differences sensitivity analysis with cohort restricted to sanctions starting in 2003 and individuals without any prison or probation time in the pre-treatment period.** Figure shows event study coefficients and their 95% confidence intervals, representing the average treatment effect on the treated (ATT) over the follow-up. ATTs for medical outcomes represent effects in percentage points. ATT for crime represents the effect in the number of charges/suspicions by the police. X-axis represents relative time to period (quarter year) where treatment first changes (time 0).


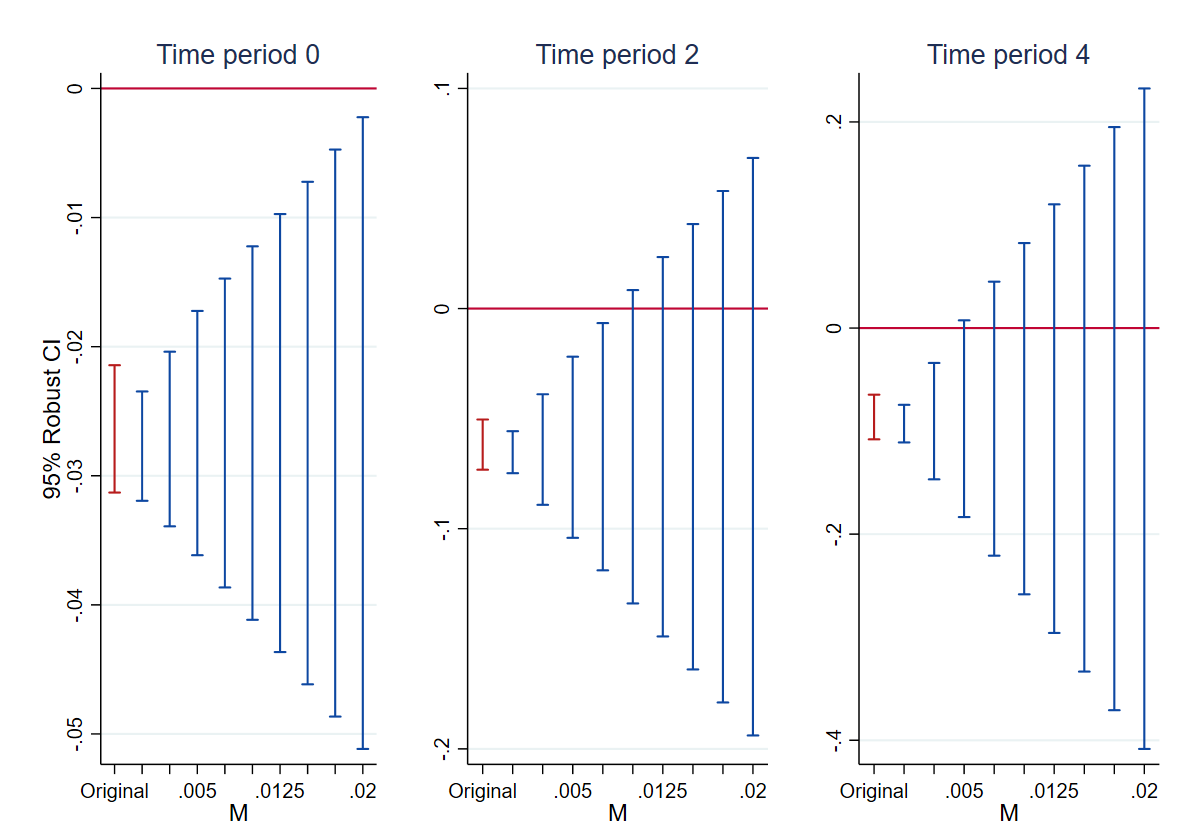


**Figure S5. Robust inference sensitivity analysis for substance misuse.** Figure shows robust confidence intervals of event study coefficients at t0, t2, and t4 calculated for different values of M. The breakdown value was 0.01 for t2, and 0.005 for t4.


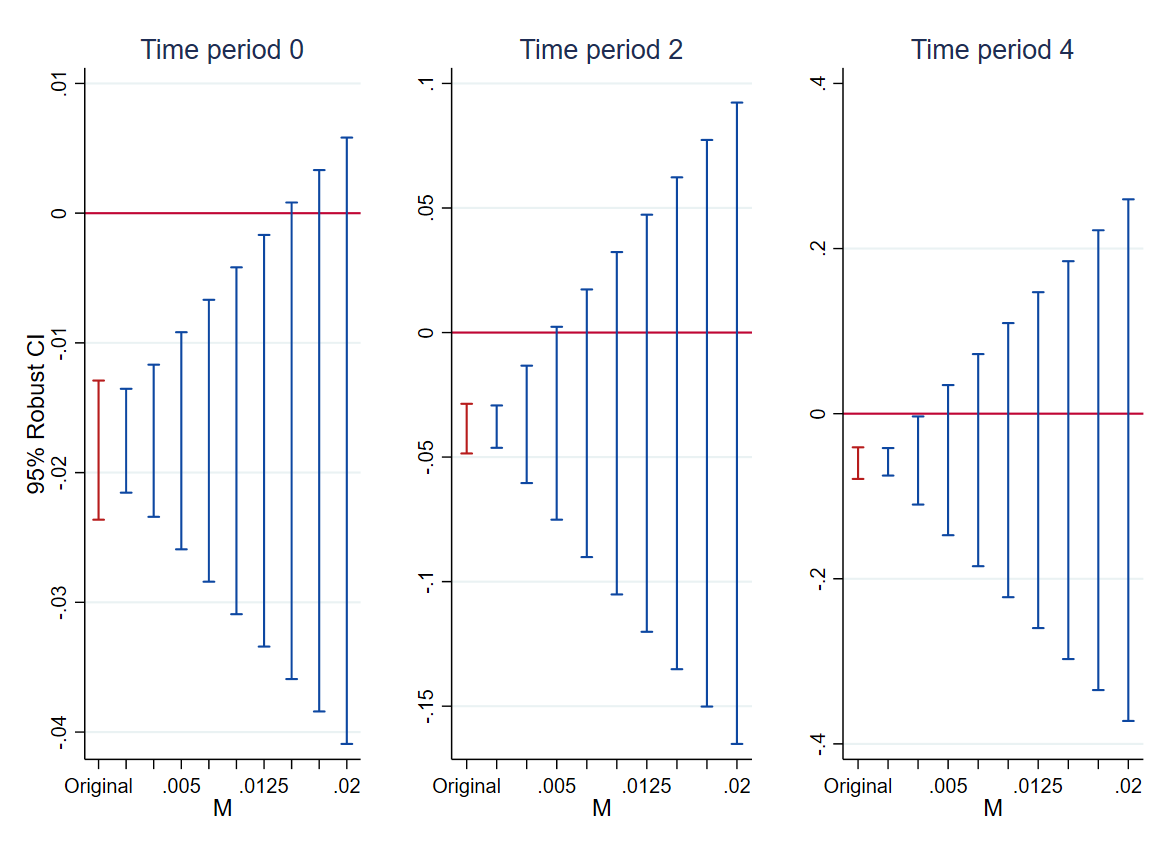


**Figure S6. Robust inference sensitivity analysis for mental health.** Figure shows robust confidence intervals of event study coefficients at t0, t2, and t4 calculated for different values of M. The breakdown value was 0.015 for t0, and 0.005 for both t2 and t4.


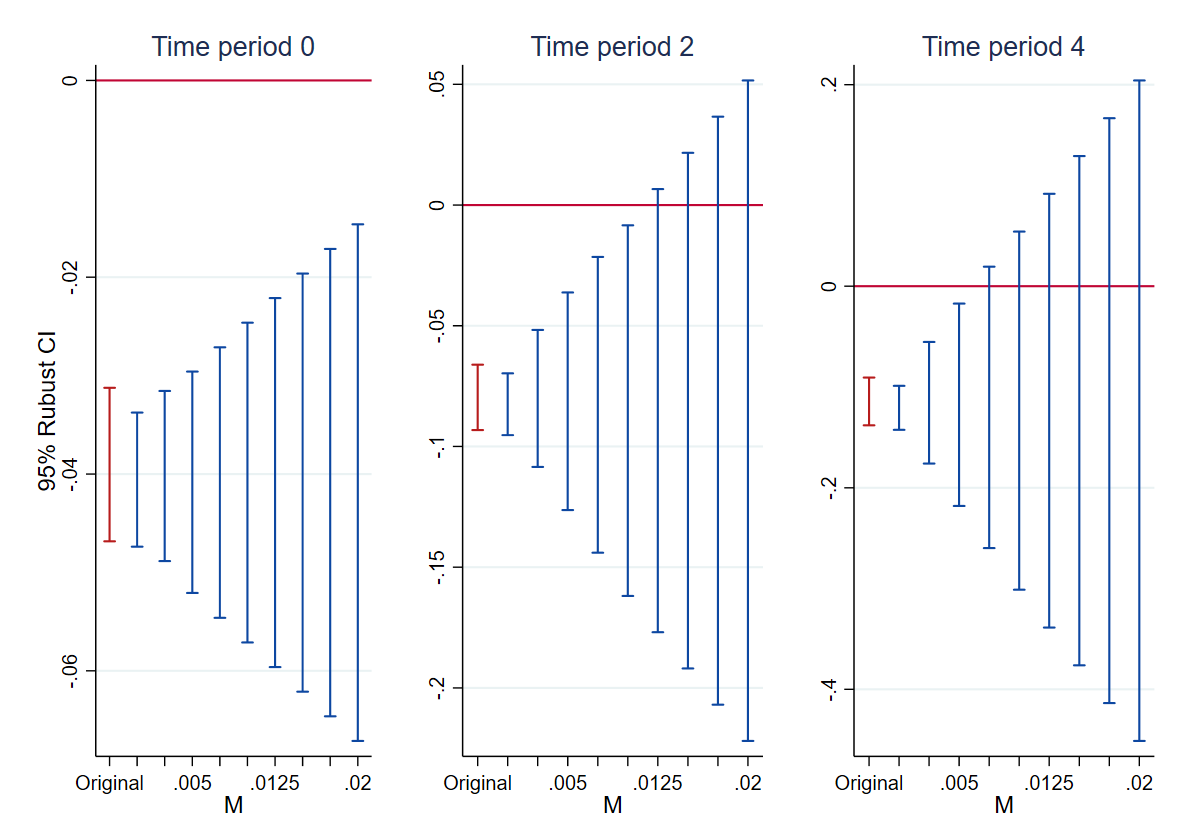


**Figure S7. Robust inference sensitivity analysis for somatic health.** Figure shows robust confidence intervals of event study coefficients at t0, t2, and t4 calculated for different values of M. The breakdown value was 0.0125 for t2, and 0.0075 for t4.


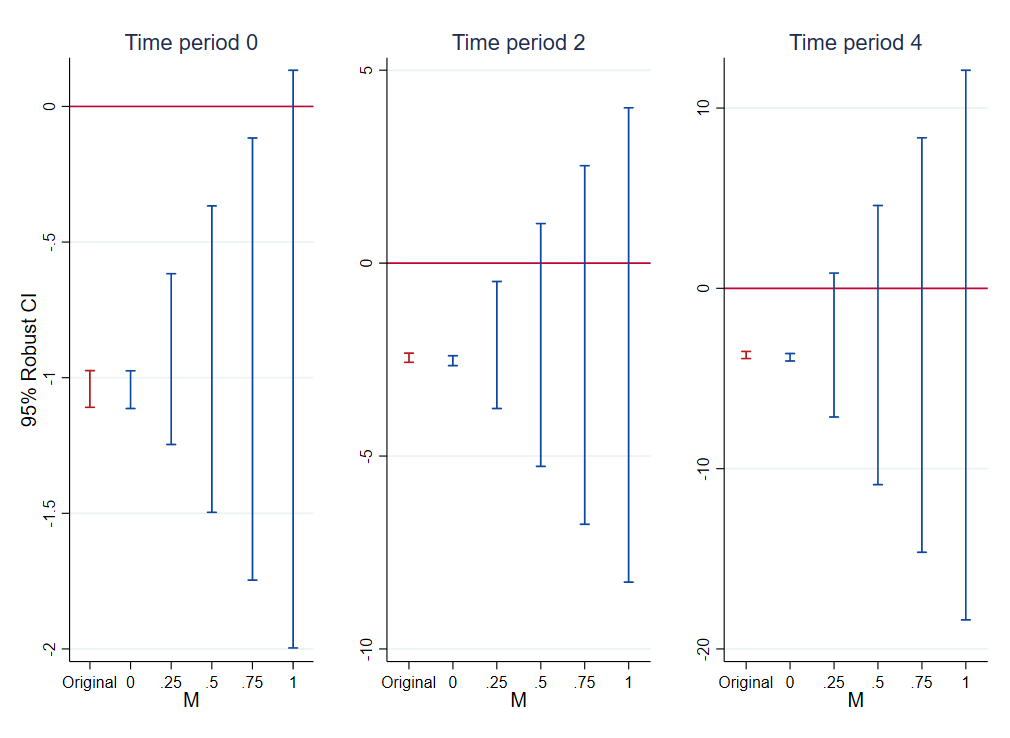


**Figure S8. Robust inference sensitivity analysis for crime.** Figure shows robust confidence intervals of event study coefficients at t0, t2, and t4 calculated for different values of M. The breakdown value was 1 for t0, 0.5 for t2, and 0.25 for t4.
